# Green immobilization and efficient proliferation of *Escherichia coli* cells in polyvinyl alcohol hydrogel membranes by unidirectional nanopore dehydration

**DOI:** 10.1186/s12934-022-01995-y

**Published:** 2022-12-19

**Authors:** Zhi-Hao Zhong, Yu-Qing Zhang

**Affiliations:** School of Biology and Basic Medical Sciences, Medical College, Soochow University, RM702-2303, No. 199, Renai Road, Industrial Park, Suzhou, 215123 China

**Keywords:** *Escherichia coli*, Immobilization, Polyvinyl alcohol, Unidirectional nanopore dehydration, Hydrogel, Proliferation

## Abstract

**Background:**

The immobilized technology for microbial or cells has the advantages of high microbial activity, high microbial density per unit space, good tolerance, strong shock, load resistance, high processing efficiency, and high reuse rate. It is now widely used in environmental remediation, water quality treatment, biodegradation, food industry, chemical analysis, energy development, medicine and pharmaceuticals, and other fields.

**Results:**

A novel *Escherichia coli* cell-immobilizing polyvinyl alcohol hydrogel membrane (ECI-PVAHM) was prepared by unidirectional nanopore dehydration (UND) from a 10% polyvinyl alcohol (PVA) aqueous solution containing enhanced green fluorescent protein-labeled *E. coli*. This bacteria-loaded film has high water stability, flexibility, transparency, and mechanical robustness. Its tensile strength, elongation rate, and swelling rate are in the ranges 0.66–0.90 MPa, 300–390%, and 330–800%, respectively. The effective bacterial load of ECI-PVAHM is 2.375 × 10^9^–10^10^ CFU/g (dry weight), which does not affect the original crystal structure of the PVAHM. This biofilm has a porous network structure with pore sizes between 0.2 and 1.0 μm, and these cells are embedded in the PVAHM network. When the immobilized cells were continuously cultured for 20 days, and the medium was renewed twice daily, their relative proliferation efficiency after 40 cycles could still be maintained at ~ 91%.

**Conclusion:**

The above results show that the cell division, proliferation ability, and metabolic activity of immobilized *E. coli* were not affected by the physical barrier of the porous network structure of the hydrogel. This UND-based ECI-PVAHM has potential applications in molecular biology, biopharmaceutical expression and production, bioreactors, and fuel cells.

## Introduction

As early as 1959, Hattori and Furusaka first immobilized *Escherichia coli* on a resin support [1]. This immobilized microorganism technology was developed based on immobilized enzymes. Using physical or chemical means, free microorganisms or cells with specific physiological or biological activity functions are immobilized in the interior or surface of the carrier material so that the immobilized microorganisms can survive and be effectively utilized as a new type of sustainable organism engineering technology [2]. This technology has the advantages of high microbial activity, high microbial density per unit space, good tolerance, strong shock, load resistance, high processing efficiency, and high reuse rate [3]. These unique advantages mean that immobilized microbial technology is now widely used in environmental remediation, water quality treatment, biodegradation, food industry, chemical analysis, energy development, medicine and pharmaceuticals, and other fields.

The carrier materials used for microbial immobilization mainly include natural and artificial polymers. Synthetic polymers, such as polyacrylamide or polyvinyl alcohol (PVA), have better mechanical stability and biodegradation than natural polymers [4]. In particular, PVA has a low price, good hydrophilicity, high mechanical strength, strong chemical stability, non-biodegradability, and no biological toxicity. It has been widely used as a carrier material for immobilizing microorganisms for a long time. PVA cryogels have become increasingly popular as cell immobilization carriers due to their very high operational stability. The properties of this polymer material were studied and described as early as the 1960s and 1970s [5–7]. The main mechanism of PVA freeze-thawing gel formation was not recognized until the 1980s [8], making it a very promising alternative to traditional gel carriers [9–11]. This PVA freeze–thaw hydrogel can immobilize glutamicum cells for L-lysine production [12] and bacillus cells for β-cyclodextrin production from agarose [13]. Many bacterial, yeast, and fungal cultures have been immobilized in this type of matrix [14–17].

However, cell immobilization in this cryogel carrier is not a simple process. The cells suspended in the PVA solution have to undergo repeated freezing and thawing, and the viscosity and water swelling of the PVA are not conducive to the preparation of an immobilized carrier. Therefore, it is necessary to add some compounds (e.g., salt, sugar, or cryoprotectant) to improve the solubility during the preparation of the PVA matrix to protect or improve the survival rate of cells. The addition of sodium alginate, powdered activated carbon, silica, zeolite powder, and other substances has improved the mechanical properties and adhesion of PVA [18]. These additives must neither interfere with PVA low-temperature gelation nor affect the physical properties of the PVA matrix. Therefore, the PVA hydrogel used to immobilize microorganisms, cells, and other active organisms should not only maintain a certain mechanical strength but also facilitate the survival of cells during the preparation process. The hydrogel network pore size and the greening and controllability of the entire preparation process must also be considered. Much research is still needed to develop an ideal PVA hydrogel for the immobilization of microorganisms.

This experiment utilizes the unidirectional nanopore dehydration (UND) technology newly developed by our team [19] to immobilize *E. coli* cells labeled with an enhanced green fluorescent protein (EGFP) in PVA hydrogel membranes and systematically investigates and discusses the physicochemical properties, mechanical properties, viability, and stability of the immobilized bacteria.

## Materials and methods

### Experimental materials and strains

PVA-124 (viscosity: 54–66 mPa s) was purchased from China Aladdin Co., Ltd.; cellulose semipermeable membrane (CO MW 14 kDa) was purchased from Viskase (USA). An EGFP-labeled construct was generated using the pUC18 vector and transformed into *E. coli* (D24831), and expression was induced with the addition of isopropyl-β-d-thiogalactoside (IPTG) purchased from Shanghai Bioresource Collection Center (SHBCC, Shanghai Bioresource Collection Center).

### The preparation of PVA aqueous solution

First, 10 g of PVA particles were slowly added to 100 mL of deionized water and heated to above 60 °C, stirring with a magnetic stirrer (500–650 rpm) until the PVA particles swelled, dissolved, and next dispersed uniformly in the water. Then, when the suspension was autoclaved at 121 °C for 20 min, the PVA was fully dissolved, forming a 10% PVA solution.

### *E. coli* culture

For the preparation of the LB (Luria–Bertani) liquid medium, 10 g/L tryptone, 5 g/L yeast extract, and 10 g/L sodium chloride were mixed with water, their pH was adjusted to 7.4 with dilute acid or a dilute alkali solution, and they were placed under high pressure at 121 °C to sterilize for 20 min. The solutions were allowed to cool to room temperature, and 50 μg/mL of kanamycin was added in a sterile environment. The *E. coli* strains were taken out of the − 80 °C refrigerator, thawed quickly, inoculated into 3 mL of LB medium with an inoculating loop, and incubated overnight at 37 °C after shaking at 220 rpm to saturation.

When an optical density at 600 nm (OD_600_) of *E. coli* culture solution reached 0.50 (9.5 × 10^9^ CFU), the inoculum was centrifuged at 5000 rpm for 5 min, the supernatant was removed, and the collected cells were volumed to one tenth of the original LB solution for use. According to previous reports [20, 21], the regression equation of the OD measured at 600 nm, and the corresponding concentration of the bacterial solution is *y* = 27.067*x* − 4.025. and added to a custom mold [19]. After slow drying over ~ 15 h under 25 °C and 60% relative humidity (RH), the *E. coli*-immobilized PVA hydrogel membrane (ECI-PVAHM) was obtained by unidirectional dehydration through the nanoporous film. Then, an 8-mm diameter punch was used to cut a hydrogel membrane with *ϕ* = 8 mm.

### Measuring mechanical strength

The mechanical properties of the bacteria-loaded PVAHM, including tensile strength and elongation at break, were measured with a universal material testing machine. Before measuring the tensile properties, the ECI-PVAHM was cut into uniform strips (6 × 20 mm), and the samples were placed in deionized water for more than 12 h to ensure complete swelling. Five samples from each group were measured, and the mean and standard deviation (± SD) were calculated.

### Determining swelling rate and dissolution loss rate

The bacteria-loaded disks (*ϕ* = 8 mm) with different thicknesses were weighed and then immersed in deionized water at 37 °C until fully swollen. The disks were removed hourly, the surface water was removed with filter paper, and the disk was weighed until the measurements did not change. This process was repeated five times for each group, and the swelling rate was calculated.

The ECI-PVAHM was tested for water stability. The ECI-PVAHM was weighed at room temperature and humidity, immersed in sterile water and placed in a 37 °C incubator. After equilibration, it was removed and dried with filter paper. The water attached to the dry surface was weighed, and after 2 months of incubation, it was removed and weighed again. Each group was repeated five times to calculate the dissolution loss rate.

### FTIR spectra, XRD spectra and thermal properties

Sample processing and instrumental analysis methods for Fourier transform infrared (FTIR) spectroscopy, X-ray diffraction spectra, and thermal property analysis were performed as previously described by the authors [22]. A small amount of powdered biosample was mixed with potassium bromide (KBr) and pressed into pellets, which were analyzed in a Fourier transform infrared (FTIR) spectrometer (Nicolet 6700, Thermo Fisher, USA). Powdered biosamples were subjected to X-ray diffraction (XRD) analysis on an X'pert-Pro MPD X-ray diffractometer (PANalytical, Holland Panalytica, The Netherlands). A 5.0 mg powdered biosample was weighed and placed in an SDT2960 differential thermal thermogravimetric analyzer (TA Instruments, USA) for TG, DTG and DSC analysis.

### SEM

Samples were prepared for and observed with scanning electron microscopy as previously described by the authors unless otherwise specified [23]. At the 3, 9 and 12 h during drying process, the sample shall not be taken out of the mold, and shall be directly placed in liquid nitrogen for fixation for 5 min, and then the membrane shall be taken out of the mold with tweezers in the presence of liquid nitrogen, and broken to form the fracture surface of the membrane, and finally freeze-dried. Take out the cracked section sample and fix it on the sample stage, then spray gold on the surface for 60 s, and observe the micromorphology of the surface and cross section of biosamples under scanning electron microscope (Carl ZEISS EVO 18, Germany) or Hitachi S-4700 cold field emission scanning electron microscope (FEI).

### Fluorescent protein expression test

In order to measure the viability and proliferation activity of immobilized *E. coli* in PVAHM, the proliferation of the immobilized bacteria was directly observed and photographed with a fluorescence microscope. The bacteria-loaded PVAHM was cultured in the medium for 12 h, and the expression of the EGFP was induced over 4 h by adding an IPTG (100 mM) inducer and photographed directly.

### Statistical methods

The data obtained in the experiment were processed with Origin 2022. Three to five replicate samples were prepared for each sample, and the calculated and measured values are expressed as the mean ± SD.

## Results

### Morphology of ECI-PVAHM

**Fig. 1:**
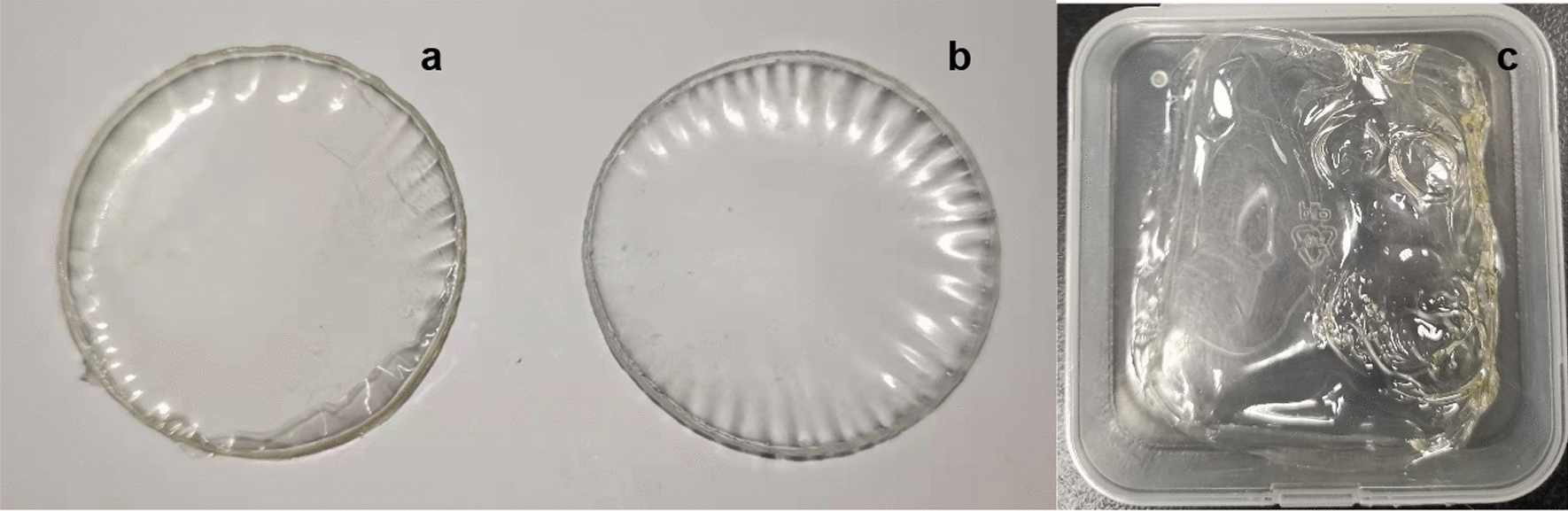
Bacteria-loaded and non-bacterial PVAHMs (dry state). **a** ECI-PVAHM (*ϕ* = 30 mm); **b** PVAHM (*ϕ* = 30 mm); **c** PVAHM prepared by a casting method (30 × 30 mm). The same volume of 10% PVA solution was casted on the plate in polypropylene box at room temperature for evaporation drying. The dried PVAHM is a casting film as a control

Figure 1 presents optical photos of three PVAHMs, the first of which is an ECI-PVAHM made using a custom UND mold (Fig. 1a). During its preparation process, 2 mL of 10 wt% PVA aqueous solution containing 0.4 mL *E. coli* solution (9.5 × 10^9^ CFU/mL) was placed in a closed space. Under the action of gravity, the water molecules permeated out from the nanopores of the bottom dialysis membrane. This process typically takes 12–24 h to dehydrate and form a film. In this experiment, to accelerate the rapid dehydration of the PVA solution, a micro fan is set to accelerate the airflow and improve the dehydration efficiency. The dry ECI-PVAHM shown in Fig. 1a has a smooth surface, uniform thickness, is hard and tough, and has a pale yellow color due to the many *E. coli* cells embedded in the film. The PVAHM of the control sample without *E. coli* was white, transparent, very tough, and hard (Fig. 1b). Another control sample was formed from 10 wt% PVA solution spread in polypropylene boxes or on plates and dried by ordinary evaporation. As shown in Fig. 1c, the formed PVA film has an uneven thickness and appears to be of poor quality.

### Swelling behavior

**Fig. 2:**
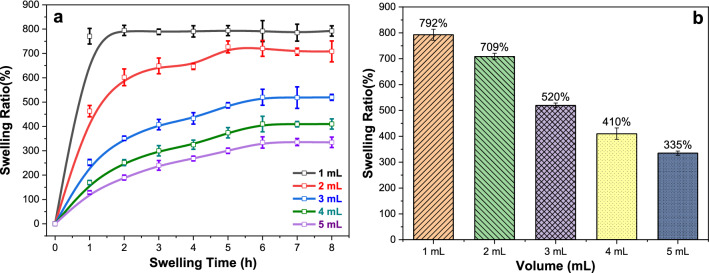
**a** Swelling curves and **b** swelling ratios of ECI-PVAHMs with different thicknesses

A hydrogel is a network of cross-linked polymer chains surrounded by an aqueous solution. Superabsorbent hydrogels have attracted extensive attention due to special advantages, such as exceptional hydrophilicity, high swelling ratio, biocompatibility, and abundant availability. This experiment simulated the effect of the environment on the swelling of PVAHMs and evaluated its applicability as a biomaterial. The results showed that all samples exhibited high equilibrium swelling ratios, while the swelling ratios of ECI-PVAHMs with different thicknesses increased rapidly in the first half of the testing period (Fig. 2a). At first, the ECI-PVAHM swollen in mL of water for 1 h approached the swelling equilibrium very quickly, which because it was the thinnest hydrogel film; the swelling ratio of the PVAHM in 2 mL reached about 450% and continued to swell; the remaining groups showed similar swelling curves, with swelling ratios between 130 and 250%. The swelling equilibrium was reached in 5 h in the 2 mL group, with a swelling rate of 700%, while the 3 mL, 4 mL, and 5 mL groups reached the swelling equilibrium in 6 h, and the final swelling rates were 510, 360 and 300%, respectively. The above results demonstrate that the UND-based bacteria-loaded PVAHM is a highly absorbent hydrogel with a swelling ratio of 300–800% (Fig. 2b). The swelling ratio of the ECI-PVAHM is inversely proportional to the thickness of the membrane. Larger amounts of PVA solution and longer periods of oriented nanopore dehydration may increase the degree of crystallization and a decrease in the porosity of the hydrogel film, resulting in decreased volume and, thus, decreased swelling rate.

### Water stability

**Fig. 3:**
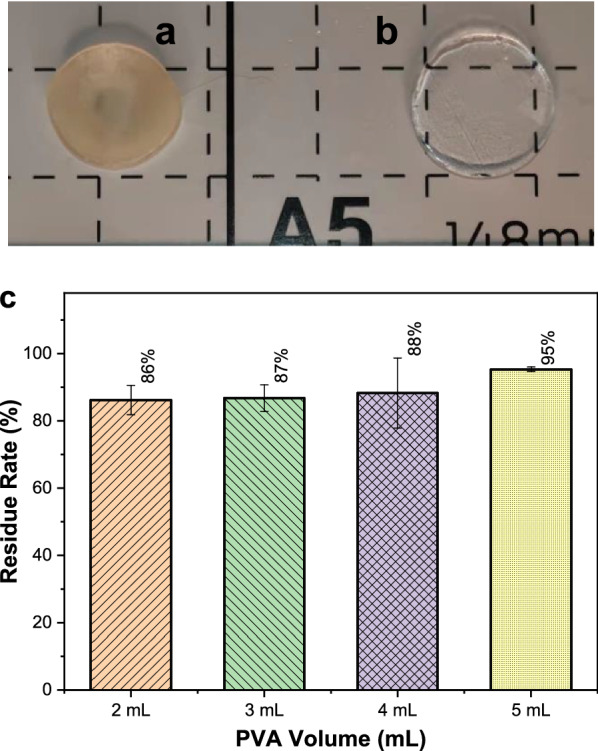
Appearance and 60-day water stability of ECI-PVAHMs loaded with bacteria in the wet state. **a**, **b** The wet bacteria-loaded and non-bacteria-loaded PVAHMs, respectively; **c** The dissolution rate experiment. The purified water immersed in the membrane was changed every 2 days, and the weight on the first day and the wet weight on the last day were used to calculate the residual amount or dissolution rate of the hydrogel film

The stability of ECI-PVAHM in aqueous medium is very important for the proliferation and growth of immobilized *E. coli*. Figure 3 is a comparison photo of the appearance and light transmittance of bacteria-loaded and non-bacteria-loaded PVAHMs. It can be seen from these images that the ECI-PVAHM is dark and almost opaque (Fig. 3a) due to the many *E. coli* cells embedded in the hydrogel film, while the ordinary PVAHM is transparent (Fig. 3b). The sizes of the two membranes are almost the same after reaching swelling equilibrium in water. Therefore, the presence of *E. coli* cells in the PVAHM did not significantly affect the swelling rate of the bacteria-loaded hydrogel film.

This bacterial-loaded PVAHM has excellent swelling properties, but its stability in water is another key factor as a 3D culture scaffold for *E. coli*. The cast PVAHM prepared by the usual method has poor mechanical properties and will stretch until the film ruptures when placed in water. Therefore, in this experiment, the non-bacteria-loaded PVAHM was placed in 25 °C water that was changed every 2 days, and the stability test was conducted for 60 days. The bar graph in Fig. 3c presents the weight loss after 2 months in water for PVAHMs of four thicknesses prepared from different volumes of PVA solution. Since the PVAHM prepared from 1.0 mL of the solution by unidirectional nanopore dehydration is too thin, in this experiment, the PVAHMs resulting from volumes of 2.0, 3.0, 4.0, and 5.0 mL were tested in water before and after 2 months. The residual amount or dissolution rate after swelling equilibrium was compared. In Fig. 3c, after 60 days of swelling in water, the dissolution loss of the first three PVAHMs with different thicknesses is 12–14%, while the thick films prepared with 5 mL of solution appear to have smaller losses (~ 5%). The difference in the dissolution loss rate of these four groups of samples, especially the average of the first three, is about 13%; it seems that the thinner PVA film has slightly higher losses. However, statistical analysis shows that there are no significant differences between the loss percentage and the volume of PVA, which may be caused by experimental error. Therefore, the hydrogel membrane is mainly composed of a hydrogen-bonded network structure, which is relatively stable and has good stability after 60 days in water.

### Mechanical tensile properties

The mechanical properties of the bacteria-loaded PVAHMs are also an important indicator. Table 1 shows the mechanical properties of the five thicknesses of ECI-PVAHMs prepared with different volumes of PVA solutions. In Table 1, the swelling rate of the PVA hydrogel film in the 1.0 mL group is very large, but the mechanical properties of the film are very poor, so it is not suitable for making biofilms. In the four experimental groups of 2.0–5.0 mL, as the volume of PVA solution used during preparation increased, the change in size after swelling did not differ greatly, and the tensile strength increased with increasing volume, except for the final 5.0 mL experimental group, which decreased. However, the elongation at break of these five groups was between 300 and 390%. The mechanical properties and elongation at break of the ECI-PVAHMs prepared with 2.0–4.0 mL of PVA solution were the best. Therefore, in subsequent biofilm preparation, unless otherwise specified, the biofilms were prepared with 2.0 mL of 10 wt% PVA aqueous solution.

**Table 1:** Tensile strength of ECI-PVAHMs prepared from different volumes of solution

PVA (mL)	After swelling (mm) width	Max load thickness	Tensile strength (*F*_max_, N)	Elongation at break (*σ*, MPa)	(%)
1.0	10.13 ± 0.50	0.34 ± 0.03	0.34 ± 0.11	0.10 ± 0.03	305.09 ± 33.76
2.0	8.38 ± 3.67	0.57 ± 0.04	2.10 ± 0.94	0.67 ± 0.64	340.52 ± 26.00
3.0	9.38 ± 0.21	0.80 ± 0.04	6.53 ± 1.37	0.88 ± 0.21	386.91 ± 19.15
4.0	9.23 ± 0.31	1.03 ± 0.08	8.36 ± 2.36	0.90 ± 0.29	359.85 ± 38.67
5.0	8.83 ± 0.35	1.25 ± 0.06	7.18 ± 1.42	0.66 ± 0.17	318.30 ± 62.74

### Stress–strain curves

**Fig. 4:**
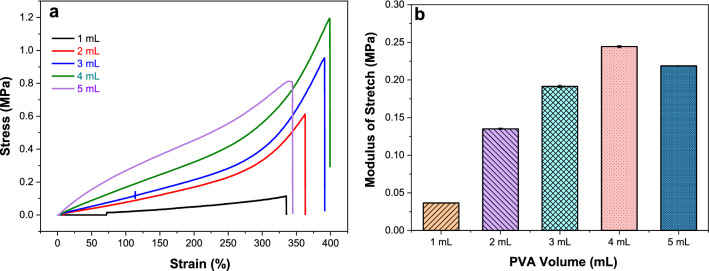
**a** Stress–strain curves and **b** tensile moduli of ECI-PVAHMs with different volumes

The mechanical properties of hydrogels are one of the key factors for evaluating their suitability for future applications. The stress–strain tensile curves of PVAHMs prepared with different volumes are notably smooth. As shown in Fig. 4a, the maximum tensile strengths of the ECI-PVAHMs of the 2.0, 3.0, 4.0, and 5.0 mL experimental groups were 0.61, 0.95, 1.2, and 0.81 MPa, respectively, while the tensile strength of the ECI-PVAHM of the 1 mL group was only 0.12 MPa. When the tensile modulus of the PVAHM was calculated using the slope of the linear elastic region of the stress–strain curve, as shown in Fig. 4b, the tensile moduli of the 2.0–5.0 mL ECI-PVAHMs were 0.14, 0.19, 0.24, and 0.22 MPa, while the tensile modulus of the 1.0 mL group of PVAHMs is only 36.55 kPa, which is far less than the mechanical properties of the other groups. This indicated that the mechanical properties of the bacteria-carrying PVAHM prepared with 1.0 mL 10% PVA aqueous solution were poor, and it cannot meet the mechanical properties required for the proliferation of *E. coli* cells. When other conditions remain unchanged, increased PVA thickness will also increase the tensile strength, reaching the maximum at 4.0 mL, while the tensile strength of the 5.0 mL group decreases. Biofilms were generally prepared with 2.0–4.0 mL of 10 wt% PVA aqueous solution.

### Efficiency and stability of cyclic proliferation

**Fig. 5:**
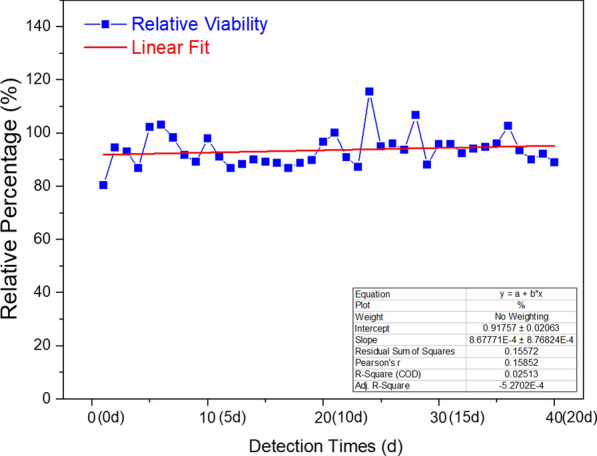
Proliferation efficiency and relative viability of immobilized *E. coli* (ECI-PVAHM) after 40 consecutive cycles of culture (20 days)

To further evaluate the viability and proliferation stability of immobilized bacteria on the porous network scaffold structure, a cyclic culture test was performed every 12 h on the bacteria-loaded PVAHM (ECI-PVAHM) to detect the proliferation and vitality of the bacteria immobilized in the membrane. The culture medium was refreshed two times each day, and the absorbance of the replaced medium was detected at 600 nm for 40 consecutive cycles, for a total of 20 days of continuous culture and proliferation tests, and the absorbance value after the first culture on the first day is referred to as 100%. The relative bacterial proliferation efficiency for each assay was calculated and plotted. As shown in Fig. 5, after 40 cycles of repeated culturing, the relative viability of the *E. coli* immobilized on the hydrogel membrane remained at about 91%. Therefore, in this experiment based on the use of UND to prepare the ECI-PVAHM, the size of the porous network and pore size can not only firmly fix the rod-shaped cells of *E. coli* but also fully ensure the original life activities, biological activities, and strong proliferative capacity of *E. coli* cells.

### Fluorescence observation of *E. coli* cells

**Fig. 6:**
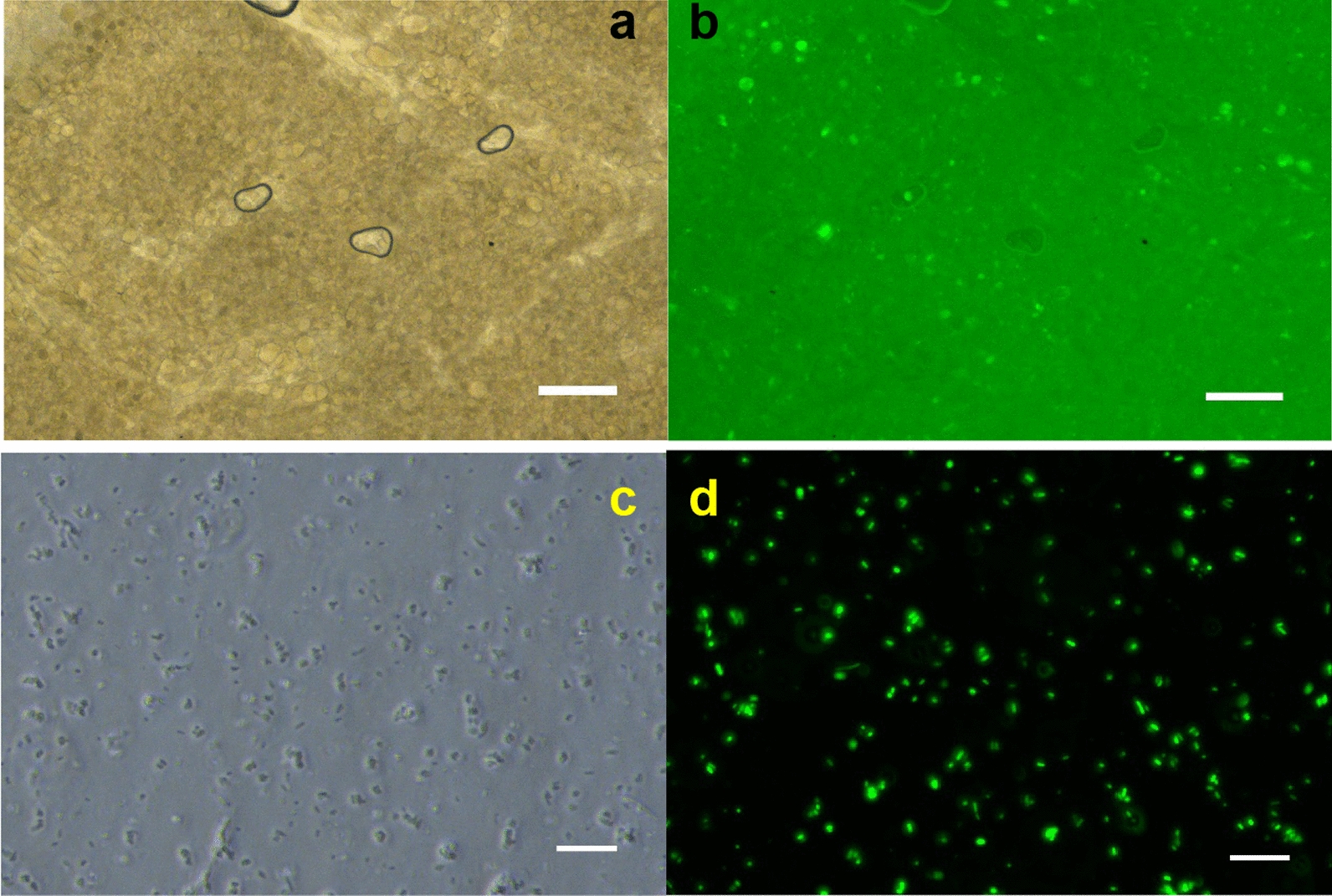
**a**, **b** Optical and fluorescent photos of ECI-PVAHM after the first 12 h cycle of culturing and **c**, **d** cells that divided and grew in the culture medium after the fortieth culture cycle. Markers: **a**, **b** 100 μm and **c**, **d** 10 μm

In this experiment, EGFP-labeled *E. coli* were used. Therefore, after repeated cell culturing and cyclic proliferation, the bacterial films were observed by sunlight and fluorescence after the first 12 h cycle of culturing and the fortieth cycle (20 days). The proliferation efficiency and viability of this EGFP-labeled ECI-PVAHM or cell can be measured. Figure 6a shows the optical photo of the bacteria-loaded PVAHM after the first 12 h culture. Under an inverted microscope with a magnification of 40×, sunlight can pass through the PVAHM, and the bacteria appear as aggregates. Under the light transmitted through the green filter, the EGFP-labeled engineering bacteria emitted a strong green fluorescence at a wavelength of 510 nm since the 0.2-mm-thick hydrogel membrane immobilized many bacteria. The green fluorescence filled the entire ECI-PVAHM (Fig. 6b). Figure 6c, d shows the *E. coli* cells that proliferated and were secure in the culture medium after the fortieth cycle (20 days) of culturing of the biofilm. Immobilized *E. coli* cells in the membrane last cell division, proliferation, and secretion of bacteria into the culture medium through the porous hydrogel network. The engineered bacterial cells and their aggregates were easily distinguished under a magnification of 400× in sunlight (Fig. 6c), while the bright green fluorescence emitted by these living engineered bacteria could be observed under a green filter (Fig. 6d). These results show that in this ECI-PVAHM network structure based on UND technology, after repeated division and proliferation of immobilized *E. coli* for 20 days, the *E. coli* remain highly active. The hydrogel network structure had few adverse effects on the life and proliferation of the cells.

### Microstructure observations

In the UND, 2.0 mL 10% PVA aqueous solution (2.0 mL) will self-assemble the PVA molecular segments into an ordered molecular structure, and the inter- and intra-chain hydroxyl groups form more and stronger hydrogen bonds[24]. After the PVA solution is added to the mold, the bottom dialysis membrane is the only connection to the atmosphere to dehydrate the water downward and reduce the water content. The speed of the final film formation mainly depends on the temperature, RH, and air velocity outside the nanofiltration membrane at the bottom of the mold during dehydration. The preparation conditions in this experiment were controlled at 25 °C and 60% RH. A micro fan was added under the mold, but it took about 12 h to dry and form a film. In order to observe the formation of the surface and internal microstructures of the PVAHM, as well as the entrapment and immobilization of *E. coli* in the hydrogel, samples were taken at two time points, 3 and 9 h before the film was completely formed. These samples were fixed in liquid nitrogen, then transferred to a vacuum freeze dryer for direct lyophilization, and the film-forming samples were processed after 12 h, according to section “Determining swelling rate and dissolution loss rate”. Finally, the surface structures of the hydrogel film and the microstructures of the longitudinal section or the fracture surface were observed.

**Fig. 7:**
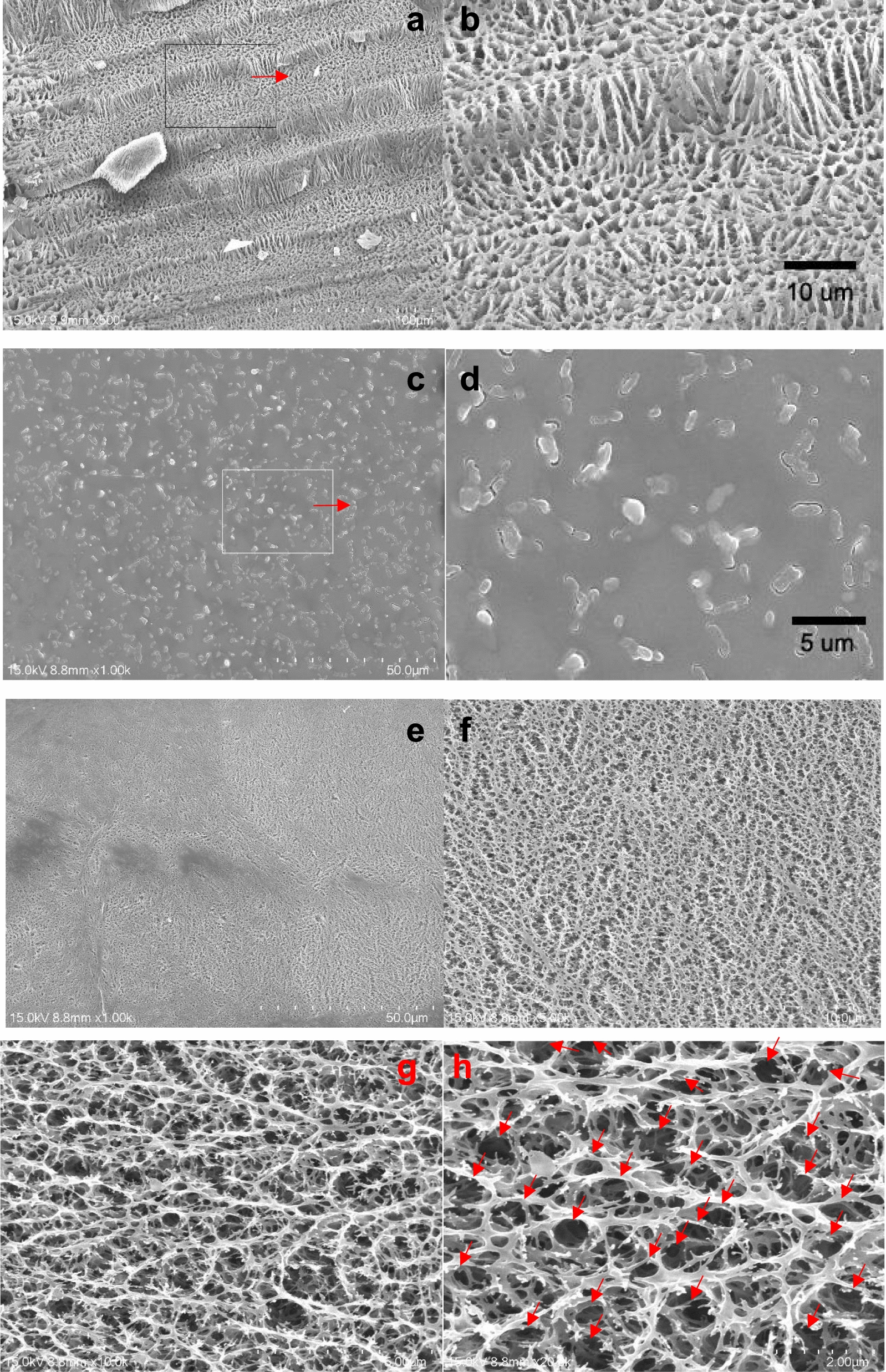
Surface and longitudinal section structures of ECI-PVAHMs during unidirectional dehydration. **a**, **b** The cross-section (3 h) at 500× and 2000×, respectively; **c**, **d** the surface of the PVA solution (9 h) at 1000× and 4000×, respectively; **e**–**h** the cross-section (12 h) at 2000×, 5000×, 10,000×, and 20,000×, respectively. The red arrows point to the *E. coli* cells embedded in the porous network

**Fig. 8:**
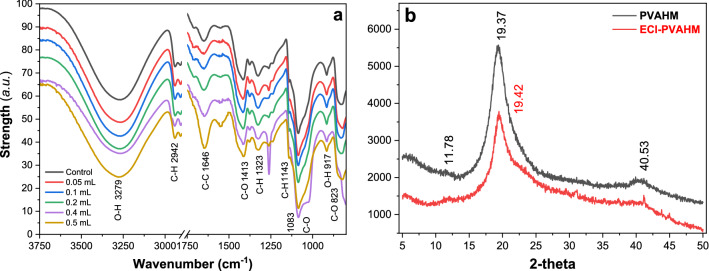
Infrared spectra (**a**) and XRD pattern (**b**) of ECI-PVAHMs. **a** 0.05, 0.1, 0.2, 0.4, and 0.5 mL of the *E. coli* solution (9.5 × 10^9^ CFU/mL), corresponding to 4.75 × 10^8^, 9.5 × 10^8^, 1.9 × 10^9^, 3.8 × 10^9^, and 4.75 × 10^9^ CFU were added, respectively in the preparation mold (7.0 cm^2^). Control: **a** PVAHM without bacteria was used as the control. **b** XRD patterns of bacteria-loaded PVAHM (4.75 × 10^9^ CFU *E. coli* cells) and PVAHM without bacteria was used as the control group

Figure 7 shows the cross-sectional SEM images of the UND 3 h before the formation of the ECI-PVAHM. Figure 8a, b are the cross-sections of the 3 h samples at 500× and 2000×, respectively. A typical neatly arranged longitudinal porous network is present, as well as a layered porous structure. The network pore size is ~ 2 μm, and the pore size will further reduce as the dehydration continues. Then, in the sample collected 9 h after dehydration, the concentrated liquid surface of the PVA aqueous solution was magnified by 1000× and observed by SEM. The dots and rod-shaped objects were almost uniform and dense. These rods are 1–2 μm in length and about 0.2–0.5 μm in diameter; these are *E. coli* bacteria embedded in the PVAHM (Fig. 7). Figure 8e, f are the cross-sectional images of the ECI-PVAHM samples (12 h) after film formation at 2000× and 5000×. After further magnification of 10,000× and 20,000× (Fig. 7g, h), the *E. coli* cells appeared embedded in the pores of the porous hydrogel network (the black part indicated by the red arrow). As a result, these ECI-PVAHMs showed a very regular porous network structure on the surface, with pores about 0.2–1 μm in diameter. These *E. coli* cells are embedded and confined in these networks and cultured from above. According to the results of the proliferation test, the fixation by these networks hardly affects the normal cell life activities of *E. coli*, and it is a suitable matrix for cell division and proliferation.

### FTIR spectra

In the FTIR spectrum of the ECI-PVAHM, the infrared absorption peak at 3279 cm^−1^ is attributed to the stretching vibration of non-bonded O–H, which reflects the degree of hydrogen bonding between the intramolecular and interchain hydroxyl groups in the PVAHM. In Fig. 8a, the PVAHMs with five different bacterial loadings (0.05, 0.1, 0.2, 0.4, and 0.5 mL) do not show obvious shifts in this absorption band, despite 4.75 × 10^−8^–10^−9^ CFU *E. coli* cells embedded and immobilized in the film. Therefore, the *E. coli* did not significantly affect the secondary structure of ECI-PVAHM. The absorption peak at 2942 cm^−1^ is assigned to the C–H stretching vibration, the absorption peak at 1143 cm^−1^ belongs to the C–H stretching vibration peak, which is related to the crystallinity of PVA [25], and the absorption peak at 1083 cm^−1^ is the C–O stretching vibration associated with the C–C stretching vibration. These vibrational peaks, that is, the antisymmetric stretching vibration peaks of O–C–C, belong to the amorphous phase. The above-mentioned infrared spectral analysis showed that the PVA hydrogel film does not significantly affect the shift of these absorption bands. This also indicates that 4.75 × 10^−8^–10^−9^ CFU *E. coli* cells should be immobilized in the porous network structure of PVA hydrogels formed by UND.

#### X-ray diffraction

The bacteria-loaded PVAHMs prepared by UND were analyzed by X-ray diffraction to determine whether the immobilized *E. coli* in the membrane affected the crystal structure of the PVAHM. The PVA hydrogel film comprises three main parts: the crystalline phase, the amorphous phase of the swollen particles, and water. The high crystallinity of the PVA hydrogel film is due to the many hydroxyl groups of the PVA molecule, and hydrogen bonds are easily formed between these hydroxyl groups within and between the chains to form a crystalline region. These results show that oriented nanopore dehydration can induce the formation of PVA hydrogel films with high crystallinity and excellent mechanical properties; more and stronger hydrogen bonds are generated between the intra- and interchain hydroxyl groups of the PVA molecules. Since the FTIR spectral analysis showed no significant differences due to different concentrations of bacteria in the membrane structure, X-ray diffraction analysis was performed only on the sample with the highest concentration of bacteria. Figure 8b shows that the PVAHM exhibited the main crystal peak of PVA at *2θ* = 19.37°, corresponding to monoclinic crystal symmetry [26]. Two smaller characteristic peaks were found at *2θ* = 11.78*°* and 40.53° [27]. After many *E. coli* were embedded and immobilized in the PVA hydrogel, the basic crystal structure of the membrane did not change significantly.

#### Thermal performance

**Fig. 9:**
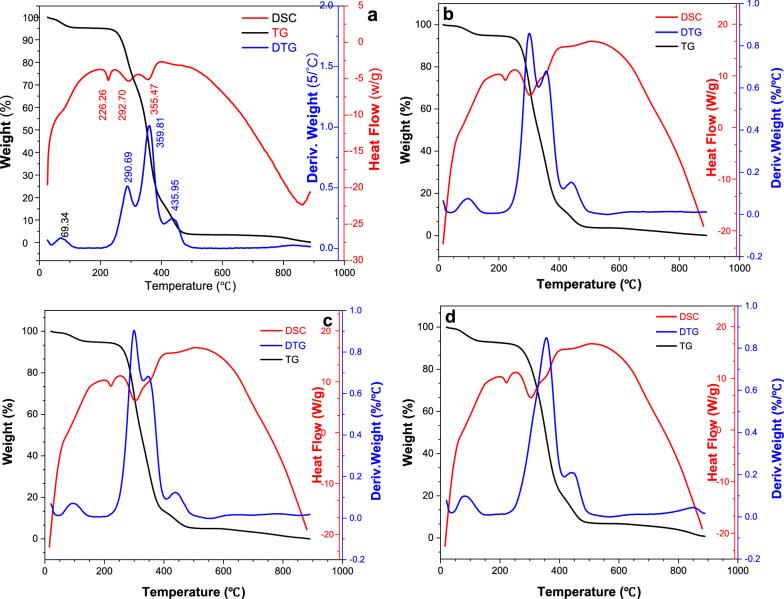
Thermal performance patterns of bacteria-loaded PVAHM. **a** 0 mL (blank group), **b** 0.05 mL, **c** 0.2 mL, and **d** 0.5 mL bacteria-loading levels in ECI-PVAHMs

PVA hydrogel is a physically cross-linked gel, and the molecular chains form microcrystalline regions through hydrogen bonds; that is, the physical cross-linking points form a three-dimensional network. These cross-linking points vary with external conditions, such as temperature. The TG curves of ECI-PVAHMs with different levels of bacteria-loading showed three similar weight loss regions [25, 28]. The first region occurred from 20 to 180 °C due to the removal and evaporation of weakly bound water; the weight loss was about 5.2%. There was no evident difference due to the number of bacteria present in these ECI-PVAHMs (Fig. 9). The second region (180–450 °C) contained two stages of weight loss and is divided into two stages. The differences between these samples were not evident in this stage, which contained three peaks on the DTG curve. The peak in the region of 50–190 °C was due to the evaporation of physically weakly bound water and chemically strongly bound water (due to relaxation in the PVA crystalline domains); the weight loss of the film was about 4.56 wt%. The second transition zone is around 260–410 °C and resulted from the degradation of the PVAHM (the melting of the crystalline domain, i.e., the breaking of hydrogen bonds); two degradation peaks appeared at this stage around 290 °C and 360 °C, and the differences between the samples were not significant. However, hydrogel membranes with a maximum bacterial load of 0.5 mL at 290 °C and 360 °C were combined into a single membrane. The peaks of the third stage of these four samples did not differ; they all occurred around 435 °C and were attributed to the main chain scission (or carbonization) of the PVA film, which is almost completely carbonized in this stage. There was no difference between these PVAHMs with or without bacterial loading in the DSC curves. Therefore, from the above thermal analysis results, the amount and presence or absence of bacteria did not influence the thermal properties of the ECI-PVAHM.

## Discussion

Bielecki and Bolek have reported that immobilizing recombinant *E. coli* in PVA gel beads made by freeze-thawing could be used for the expression of phenol lyase [29]. Recombinant *E. coli* cells and 12% PVA solution were made into a suspension, then injected into an edible oil at − 20 °C to form microbeads after five cycles of freeze–thaw treatment at − 30 °C to obtain the resulting *ϕ* = 1–2 mm bacteria-loaded PVA microbeads. Gao et al. have used compressed nitrogen gas sheared into droplets to encapsulate transgenic engineered bacterial cells expressing urease in PVA–alginate microcapsules. In vitro experiments have shown that wet bacterial cells in 100-mg PVA microcapsules can remove 18.4 mg of urea from uremic patients within 4 h [30]. Wang et al. have prepared PVA microcapsules loaded with genetically engineered *E. coli* DH5α cells using a modified physical cross-linking methodat low temperature [31]. The bacteria-loaded PVA mixed aqueous solution was suspended in lubricating oil with Span 80 as a dispersant. The entire system was subjected to the treatment process of freezing in a low-temperature water bath at − 20 °C for 18 h and − 2 °C for 24 h before thawing to room temperature. Thus, bacteria-loaded PVA microcapsules were obtained. A macroporous polymer scaffold has been obtained by the unidirectional freezing (− 196 °C) of PVA buffered aqueous solution. Then it was placed in the *E. coli* culture medium for in situ immobilization of *E. coli*, thereby forming a PVA scaffold carrying bacteria [32]. Most of the above methods are based on multiple freeze–thaw cycles of the PVA aqueous solution or multiple freeze–thaw cycles after directional freezing to form engineered bacteria encapsulated or entrapped in PVA hydrogels, particles or capsules. The immobilization process of the above-mentioned engineered bacteria is difficult to control, and the alternating cycle of high and low temperature does not preserve the vitality of the engineered bacteria. At the same time, the cycled catalytic efficiency or proliferation efficiency of these immobilized bacteria has rarely been investigated. Rebroš et al. have described the immobilized cyclopentanone monooxygenase-overexpressing *E. coli* BL21(DE3) in PVA gel particles for biocatalysis [33]. In a drying cabinet with air circulation, it is injected into the hard material panel through a small nozzle to form a PVA gel particle in which the engineered bacteria are embedded and then swells in a special stabilizing solution to become an immobilized *E. coli* gel particle. The catalytic efficiency was maintained after six reaction cycles, whereas the catalytic efficiency of the free cells decreased after three reaction cycles.

In this research, a suspension of 10% PVA in aqueous solution and EGFP-labeled *E. coli* cells was unidirectionally dehydrated through filter nanopores for 12 h under aseptic conditions at room temperature to obtain a mechanically strong (0.66–0.90 MPa) and translucent PVA hydrogel film with good extensibility (300–390%), a swelling ratio of up to 700%, and an effective bacterial load of 2.375 × 10^9^–10^10^ CFU/g (dry weight). This ECI-PVAHM has a porous network structure with a pore size between 0.2 and 1.0 μm. The *E. coli* cells are embedded and immobilized in the porous network of the PVA hydrogel membrane, allowing the oxygen transport necessary for cell survival. The removal of wastes secreted by cells and nutrient transport did not affect the division and proliferation of the *E. coli* cells.

The bacteria-loaded PVAHM based on UND technology was continuously cultured by shaking for 40 cycles for 20 days, and the culture medium was replaced every 12 h. The absorbance value of each culture medium, the proliferation efficiency, was measured twice per day, and the relative proliferation efficiency was calculated with the initial measured value of 100%. After 20 days (40 cycles), the relative proliferation activity remained at about 91%. In the hydrated state, this hydrogel has diffusion transport properties similar to that of a liquid, which ensures that nutrients can diffuse freely throughout the entire scaffold structure to meet the needs of bacterial proliferation and growth. The proliferation ability and metabolic activity of the immobilized *E. coli* remained largely unchanged for 20 days. The mechanical strength, stretchability, swelling property, and water stability of this porous network structure biofilm could support repeated proliferation, long-term culturing, and the stability of immobilized microorganisms. Moreover, the preparation method is green, low-carbon, simple, has a controllable immobilization process, and has potential applications and development value in the huge demand for the expression of engineered bacteria; for example, biopharmaceuticals, which increases day by day.

## Data Availability

The datasets used and/or analyzed during the current study as well as analysis scripts are available from the corresponding author on reasonable request.
